# Single-gene knockout-coupled omics analysis identifies C9orf85 and CXorf38 as two uncharacterized human proteins associated with ZIP8 malfunction

**DOI:** 10.3389/fmolb.2022.991308

**Published:** 2022-10-18

**Authors:** Heng Wee Tan, Yan-Ming Xu, Zhan-Ling Liang, Na-Li Cai, Yu-Yao Wu, Andy T. Y. Lau

**Affiliations:** Laboratory of Cancer Biology and Epigenetics, Department of Cell Biology and Genetics, Shantou University Medical College, Shantou, Guangdong, China

**Keywords:** C9orf85, CXorf38, iTRAQ, proteomics, ZIP8

## Abstract

Human transmembrane protein metal cation symporter ZIP8 (SLC39A8) is a member of the solute carrier gene family responsible for intracellular transportation of essential micronutrients, including manganese, selenium, and zinc. Previously, we established a ZIP8-knockout (KO) human cell model using the CRISPR/Cas9 system and explored how the expression of ZIP8 could possibly contribute to a wide range of human diseases. To further assess the biophysiological role of ZIP8, in the current study, we employed isobaric tags for relative and absolute quantitation (iTRAQ) and detected the changes of the proteome in ZIP8-KO cells (proteomic data are available *via* ProteomeXchange with identifier PXD036680). A total of 286 differentially expressed proteins (206 downregulated and 80 upregulated proteins) were detected in the ZIP8-KO cell model, and subsequent bioinformatics analyses (GO, KEGG, KOG, and PPI) were performed on these proteins. Interestingly, four “uncharacterized” proteins (proteins with unknown biological function) were identified in the differentially expressed proteins: C1orf198, C9orf85, C17orf75, and CXorf38—all of which were under-expressed in the ZIP8-KO cells. Notably, C9orf85 and CXorf38 were amongst the top-10 most downregulated proteins, and their expressions could be selectively induced by essential micronutrients. Furthermore, clinical-based bioinformatic analysis indicated that positive correlations between the gene expressions of *ZIP8* and *C9orf85* or *CXorf38* were observed in multiple cancer types. Overall, this study reveals the proteomic landscape of cells with impaired ZIP8 and uncovers the potential relationships between essential micronutrients and uncharacterized proteins C9orf85 and CXorf38. The differentially expressed proteins identified in ZIP8-KO cells could be the potential targets for diagnosing and/or treating human ZIP8-associated diseases, including but not limited to malnutrition, viral infection, and cancers.

## Introduction

The human ZRT/IRT-like protein 8 (ZIP8), encoded by the solute carrier family 39 member 8 gene *SLC39A8*, is a metal cation symporter located mainly on the cell membrane (Zang et al., 2016). ZIP8 is well-known for its role in transporting divalent metal ions into the cells—these ions include several essential micronutrients (e.g., iron [Fe], manganese [Mn], and zinc [Zn]) and non-essential toxic heavy metal cadmium (Cd) (Wang et al., 2012; Xu et al., 2017).

A balanced level of essential micronutrients is crucial for maintaining human health (Mezzaroba et al., 2019; Tan et al., 2019). Studies have indicated that cells with impaired ZIP8 are often associated with disrupted micronutrient homeostasis, which may lead to a wide range of human disorders (Boycott et al., 2015; Park et al., 2015). It has been documented that impaired ZIP8, usually caused by mutations in the *SLC39A8* gene, is responsible for diseases such as type II congenital disorder of glycosylation (Riley et al., 2017; Choi et al., 2018), cardiovascular diseases (Zhang et al., 2016), severe idiopathic scoliosis (Haller et al., 2018), schizophrenia (McCoy et al., 2019), Crohn’s disease (chronic inflammation of the digestive system) (Li et al., 2016), and Leigh syndrome (a severe inherited neurodegenerative disease) (Choi et al., 2018). These disease-associated *SLC39A8* gene mutations include 97G>A, 112G>C, 338G>C, 610G>T, 1004G>C, 1019T>A, and 1171G>A (Boycott et al., 2015; Park et al., 2015; Park et al., 2018).

Recently, we used CRISPR/Cas9 genome editing technology to knockout (KO) the *SLC39A8* gene in the human cervical cancer HeLa cell line (Liang et al., 2021). We then studied the micronutrient transport ability of the single-gene KO cell model and discovered that the elimination of ZIP8 could result in not only the reduced cellular uptake of the above-mentioned metals, but also reduced uptake of another essential micronutrient, selenium (Se) (Liang et al., 2021). Furthermore, by utilizing clinical datasets of 40 different types of cancers from The Cancer Genome Atlas (TCGA) database, we showed that *SLC39A8* gene expressions tend to be upregulated in a great number of tumor types (Liang et al., 2021). This data suggested that ZIP8 could be a novel molecular target in preventing or treating human cancers and/or illnesses related to Cd exposure. However, the proteome in cells with impaired ZIP8 has remained uninvestigated.

In this study, we sought to better elucidate the biophysiological role of ZIP8 by carrying out a single-gene KO-coupled omics (SGKOmics) analysis on our established ZIP8-KO cell model. Specifically, proteomes of the established ZIP8-KO and ZIP8-wildtype (WT) cell lines were quantified using isobaric tags for relative and absolute quantitation (iTRAQ) and liquid chromatography-tandem mass spectrometry (LC-MS/MS). Differentially expressed proteins between the two cell lines were identified, and subsequent bioinformatics analyses were performed. Among the differentially expressed proteins, four were recognized as the “uncharacterized proteins” with unknown biological functions: C1orf198, C9orf85, C17orf75, and CXorf38. Notably, C9orf85 and CXorf38 were two of the top-10 most downregulated proteins. It has so far remained unknown whether the expressions of C9orf85 and CXorf38 could be affected by ZIP8-transportable essential micronutrients (e.g., Mn, Se, and Zn), and therefore, we further examined the expressions of these two uncharacterized proteins upon essential micronutrient treatments. In addition, we performed clinical-based bioinformatic analysis to evaluate the potential connections between the gene expressions of *ZIP8* and *C9orf85* or *CXorf38* in multiple cancer types. Overall, findings from this study provide insights into the underlying mechanisms of ZIP8 deficiency-associated diseases by indicating that cell line with impaired ZIP8 contains an aberrant protein profile, which may be due in part to imbalanced levels of intracellular micronutrients.

## Materials and methods

### Cell lines and culture conditions

Human cervical cancer (HeLa) cell line was purchased from the American Type Culture Collection (ATCC) (Rockville, MD, United States). A ZIP8 single-gene KO HeLa cell model was established using the CRISPR/Cas9 genome editing technology, as described in Liang et al. (2021). All cell lines used in the current study were routinely cultured in MEM medium containing 10% fetal bovine serum and 1% penicillin/streptomycin at 37°C in a 5% CO_2_ incubator as recommended by ATCC.

### Protein sample preparation

HeLa parental cell line (with ZIP8-WT) and ZIP8-KO HeLa cell line were grown to 80%–85% confluency (approximately 1 × 10^7^ cells per sample) in 10 cm diameter Petri dishes prior to protein extraction. Samples ready to be extracted were placed on ice and washed with 5 ml pre-chilled phosphate-buffered saline (PBS) for six times. Then, 1 ml PBS per dish was added, and cells were scratched with a sterile plastic scraper. The cell suspension was then transferred to a 1.5 ml Eppendorf tube and centrifuged at 1,200 × *g* for 5 min at 4°C. After centrifugation, the supernatant was carefully removed, and the sample was stored at −80°C.

### Protein extraction and digestion for iTRAQ analysis

Extraction and digestion of protein were performed by Wininnovate Bio Co., Ltd (Shenzhen, China). Briefly, frozen cell samples were lysed in RIPA lysis buffer (0.1% SDS, 1% Triton X-100, 150 mM NaCl, 1 mM EDTA, 0.5 mM EGTA, and 50 mM Tris-HCl pH 7.4) containing PhosSTOP^™^ protease inhibitor cocktail. The samples were subsequently homogenized by sonication on ice with 15% ultrasound power under the repeated settings of “2 s on and 3 s off” for 4 min. The homogenate was then cleared using centrifugation at 12,000 rpm for 15 min at 4°C. Next, supernatants were transferred into clean tubes, and protein concentrations were determined using the Pierce^™^ BCA Protein Assay Kit. For protein digestion, 150 µg of protein from each lysate was mixed with solutions of 8 M urea, 0.1 M Tris-HCl, 0.1 M dithiothreitol, and 5 mM iodoacetamide, as described in Wiśniewski et al. (2009). Nanosep^®^ centrifugal devices were used to centrifuge the samples.

### iTRAQ labeling and LC-MS/MS analysis

iTRAQ and LC-MS/MS analysis were performed by Wininnovate Bio Co., Ltd (Shenzhen, China). Briefly, samples were labeled with iTRAQ reaction reagents according to the manufacturer’s instructions. Each iTRAQ reagent was dissolved in 70 μl of isopropanol and added to the respective peptide mixture for 120 min. The labeling reaction was quenched by the addition of 100 μl of Milli-Q^®^ H_2_O, and all labeled samples were then pooled into one sample. The ZIP8-WT control samples (H1, H2, and H3) were labeled with tag-113, -114, and -115, while the ZIP8-KO samples (Z1, Z2, and Z3) were labeled with tag-116, -117, and -118.

LC-MS/MS detection was performed using data-dependent acquisition (DDA) MS techniques on a Thermo Scientific^™^ Q Exactive^™^ MS fitted with a Nanospray Flex^™^ ion source. Data was acquired using an ion spray voltage of 1.9 kV and an interface heater temperature of 275°C. The MS was operated with FULL-MS scans. For DDA, survey scans were acquired in 250 msec, and up to 20 product ion scans (50 msec) were collected. Only spectra with a charge state of 2–4 were selected for fragmentation by higher-energy collision. The MS/MS data were analyzed for protein identification and quantification using the Proteome Discoverer^™^ (v2.1.0.81). The local false discovery rate was 1.0% after searching against the *Homo sapiens* protein database with a maximum of two missed cleavages and one missed termini cleavage. The following settings were selected: Oxidation (M), Acetylation (Protein N-term), Deamidation (NQ), Pyro-glu from E, and Pyro-glu from Q for variable modifications; Carbamidomethylation (C), iTRAQ 8plex (K), and iTRAQ 8plex (Peptide N-term) for fixed modifications. Precursor and fragment mass tolerance were set to 10 ppm and 0.05 Da, respectively. The MS proteomics data have been deposited to the ProteomeXchange Consortium *via* the PRIDE (Perez-Riverol et al., 2022) partner repository with the dataset identifier PXD036680 (10.6019/PXD036680).

### Bioinformatics analysis

For ZIP8-WT and ZIP8-KO samples, two of the most closely related biological replicates were selected (tag-114, -115, -117, and -118) for further bioinformatic analysis. Proteins with *p* ≤ 0.05 and expression fold-change ≥ 1.2 or ≤ −1.2 were considered differentially expressed, and these proteins were compared by a hierarchical cluster analysis using hclust function (complete linkage method) in R software. All differentially expressed proteins were then subjected to Gene Ontology (GO) enrichment, Kyoto Encyclopedia of Genes and Genomes (KEGG) pathway, Eukaryotic Orthologous Groups (KOG) annotation, and subcellular localization analyses. The KOG annotation analysis was performed using automated construction and annotation of orthologous groups of genes (eggNOG) database. Subcellular localization of proteins was analyzed using Bologna Unified Subcellular Component Annotator (BUSCA). Protein-protein interaction (PPI) network analysis was carried out on all or specifically selected differentially expressed proteins using the STRING database and visualized using Cytoscape 3.9.1; the minimum required interaction score was set at 0.4 medium confidence. Clinical-based bioinformatic analysis was performed to assess the co-expression of *ZIP8* and top-10 upregulated or downregulated ZIP8-KO-associated protein genes in 40 cancer types; co-expression data was obtained from TCGA database and analyzed by Spearman’s correlation.

### Immunoblot analysis

HeLa cells or ZIP8-KO cells were treated with various concentrations of Mn, Se, or Zn for 24 h prior to immunoblot analysis. Specifically, MnCl_2_, Na_2_SeO_3_, or ZnCl_2_ were dissolved directly in MEM medium and sterilized with 0.45 μm filters. Micronutrient-treated samples were then lysed with sample buffer at 95°C for 10 min. After that, cell lysates were centrifuged at 16,900 × *g* for 10 min at room temperature, and the extracted proteins were used for immunoblot analysis as described previously (Qin et al., 2020). Three antibodies were used: C9orf85 (PA5-65639; 1:1,000) and CXorf38 (PA5-62139; 1:1,000) were purchased from Invitrogen and β-actin (A5441; 1:10,000) was purchased from Sigma-Aldrich. Quantifications of immunoblot results were performed using ImageJ (v1.51j8) and GraphPad Prism^®^ 8 (v8.0.2, GraphPad Software Inc.).

### Statistical analysis

Unconstrained principal component analysis (PCA) was used to determine the distributions of protein expressions in ZIP8-WT control samples and ZIP8-KO samples. Quantitative data of immunoblotting was expressed as mean ± standard deviation (SD) of at least three replicates. Student’s t-test was used for statistical analysis between the untreated control and treated samples, and a probability of *p* ≤ 0.05 was used as the criterion for statistical significance. Statistical analysis was performed using GraphPad Prism^®^ 8 software (v8.0.2, GraphPad Software Inc.).

## Results

### Proteome of human cells with ZIP8 deficiency obtained by iTRAQ-Based comparative proteomic analysis

**FIGURE 1:**
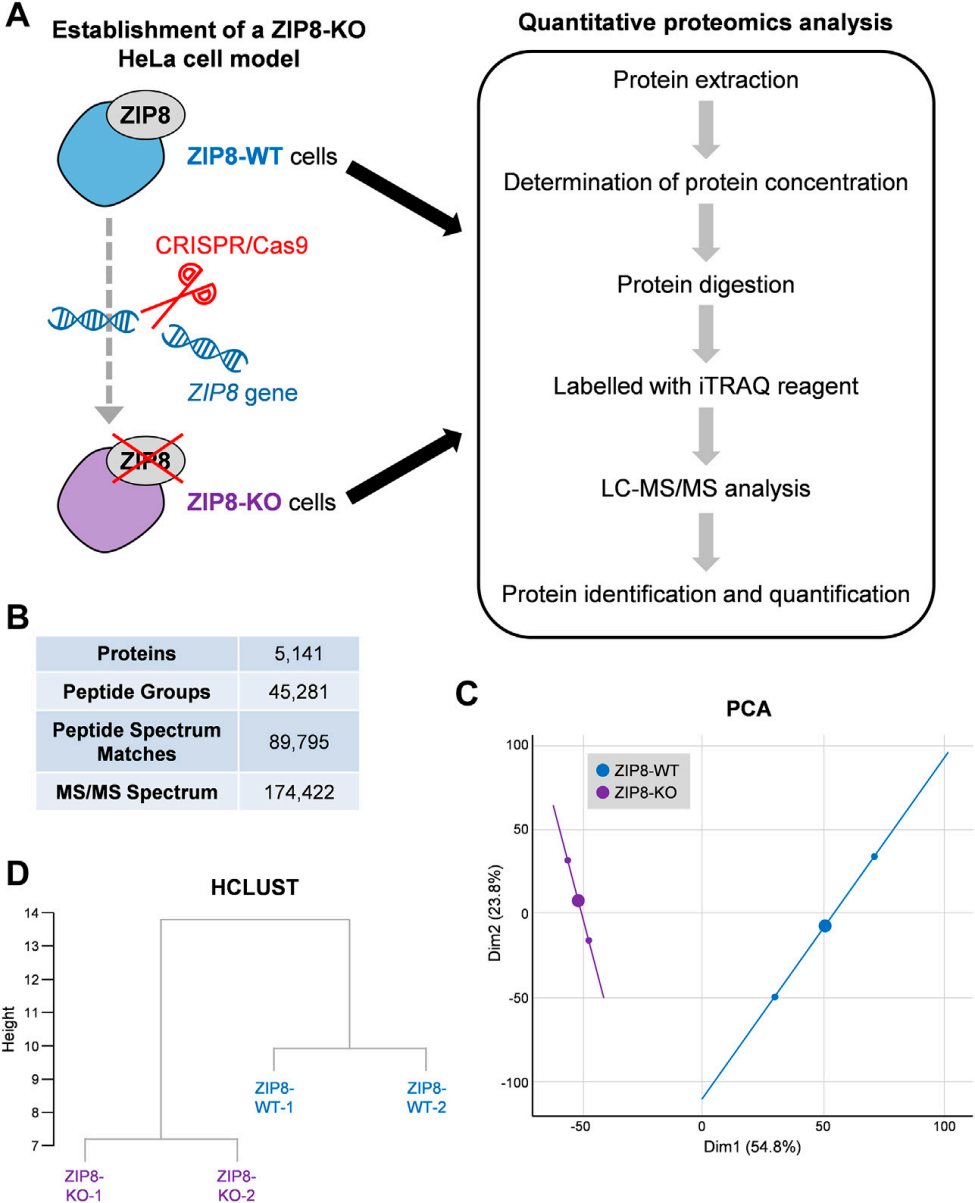
Quantitative proteomic analysis of human cells with ZIP8-wildtype (WT) or -knockout (KO). **(A)** Schematic diagram of the cell models subjected to iTRAQ-based comparative proteomic analysis in this study. **(B)** LC-MS/MS data after searching against Homo sapiens protein database for protein identification and quantification. **(C,D)** Principal component analysis (PCA) **(C)** and cluster dendrogram (HCLUST) **(D)** of all identified proteins between ZIP8-WT and ZIP8-KO cells.

We previously established a ZIP8-KO HeLa cell model using the CRISPR/Cas9 genome editing technology and showed that ZIP8 is a transmembrane protein responsible for the cellular uptake of Zn, Mn, Se, and Cd (Liang et al., 2021). Here, protein profiles of ZIP8-WT and ZIP8-KO HeLa cell lines were obtained by iTRAQ-coupled LC-MS/MS analysis (Figure 1A; Supplementary Table S1).

A total of 174,422 spectra and 45,281 unique peptides corresponding to 5,141 proteins were identified in the tested samples at a false discovery rate of 1% (Figure 1B; Supplementary Figure S1). PCA of the identified proteins showed that the ZIP8-WT and ZIP8-KO cells were generally closer to their biologically replicated counterparts (*n* = 2) but were more distant between different cell lines, indicating an overall dissimilarity of the two cell lines (Figure 1C). Also, hierarchical clustering based on Euclidean distance of the two cell lines showed similar results (Figure 1D).

### GO enrichment analysis of differentially expressed proteins in the ZIP8-KO cell model

**FIGURE 2:**
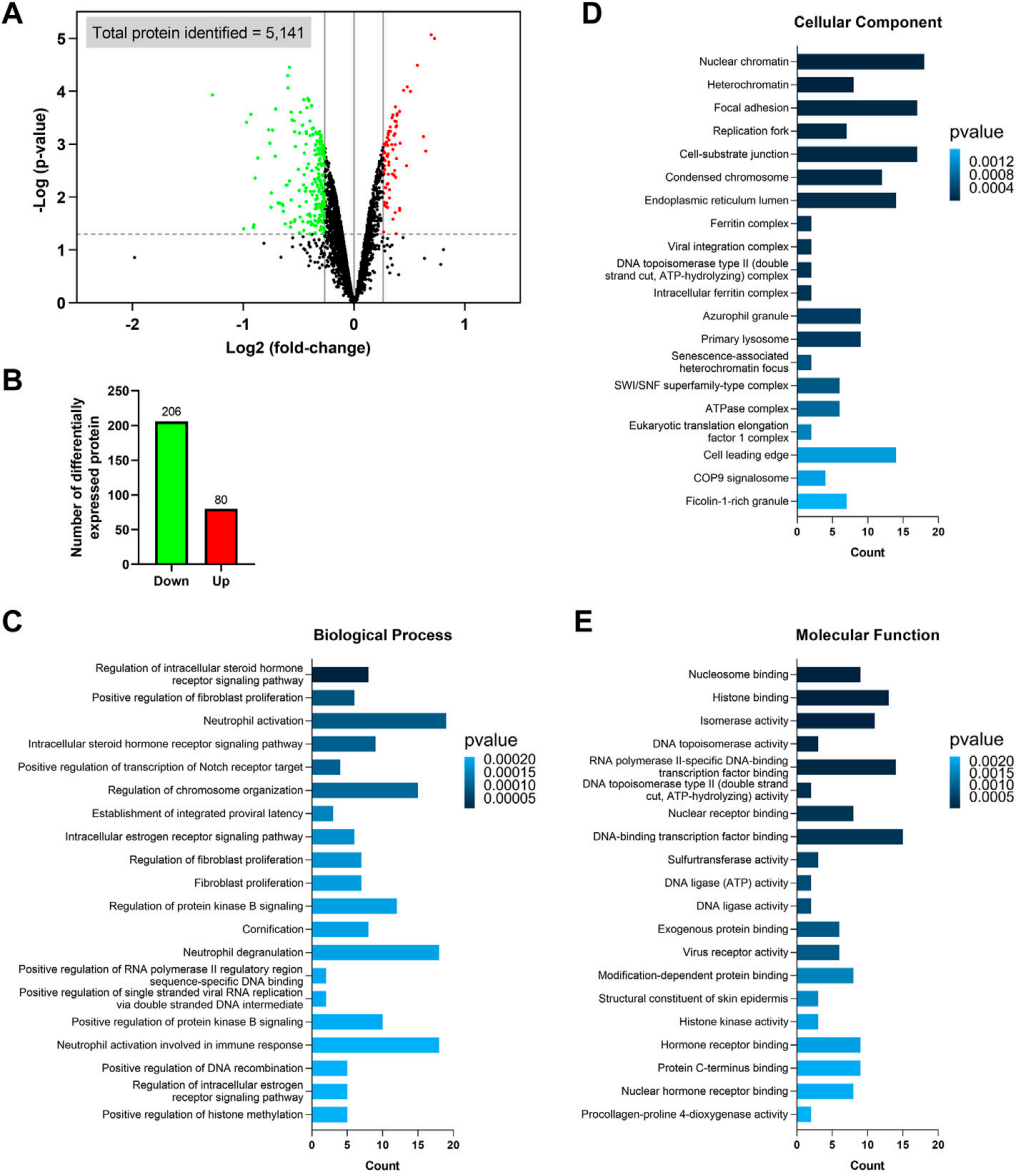
Identification of differentially expressed proteins and Gene Ontology (GO) enrichment analysis. **(A)** Volcano plot showing the differentially expressed proteins identified in the ZIP8-KO cells. Only proteins with *p* ≤ 0.05 and expression fold-change ≥ 1.2 or ≤ −1.2 are considered significant, and these proteins are highlighted in green (downregulated) or red (upregulated). Vertical lines on the x-axis represent 1.2 or −1.2 fold-change; dash line on y-axis represents *p* = 0.05. **(B)** Number of upregulated and downregulated proteins. **(C–E)** GO enrichment analysis of the differentially expressed proteins classified based on biological process **(C)**, cellular component **(D)**, and molecular function **(E)**.

Among the 5,141 identified proteins, 286 were differentially expressed between the ZIP8-KO and ZIP8-WT cells (*p* ≤ 0.05 and expression fold-change ≥ 1.2 or ≤ −1.2) (Figure 2A; Supplementary Table S1). Specifically, the ZIP8-KO cells contained 206 downregulated and 80 upregulated proteins as compared to the ZIP8-WT cells, as shown in Figures 2A,B. Noteworthily, four of the 206 downregulated proteins were classified as the “uncharacterized” proteins: C1orf198, C9orf85, C17orf75, and CXorf38.

The differentially expressed proteins were subsequently classified using the GO categories (Figures 2C–E). Overall, results indicated that the most important biological processes of these proteins were related to fibroblast proliferation, neutrophil activation and degranulation, viral RNA replication, and receptor signaling pathways involving the regulations of steroid hormone, estrogen, Notch, and protein kinase B (Figure 2C; Supplementary Table S2). For GO cellular component enrichment analysis, it was revealed that most proteins were mainly associated with chromatin/chromosome, focal adhesion, replication fork, endoplasmic reticulum lumen, primary lysosome, azurophil granule, and complexes involving viral integration, ferritin, DNA topoisomerase type II, SWI/SNF superfamily-type, ATPase, and eukaryotic translation elongation factor 1 (Figure 2D; Supplementary Table S3). The GO terms of molecular function showed that the differentially expressed proteins mostly participated in the bindings of DNA-binding transcription factor, exogenous protein, histone, hormone receptor, modification-dependent protein, nuclear hormone receptor, nuclear receptor, nucleosome, and protein C-terminus, as well as activities of DNA ligase, DNA topoisomerase, histone kinase, isomerase, procollagen-proline 4-dioxygenase, sulfurtransferase, and virus receptor (Figure 2E; Supplementary Table S4). Furthermore, since ZIP8 was best known for its role in transporting divalent metal ions, we also looked at which GO categories related to “transporter regulation,” “metal response,” and “stress response” were significantly enriched, and these categories, along with their associated differential expressed proteins, were summarized in Supplementary Figure S2.

### KEGG pathway, eggNOG annotation, and subcellular localization analyses of differentially expressed proteins

**FIGURE 3:**
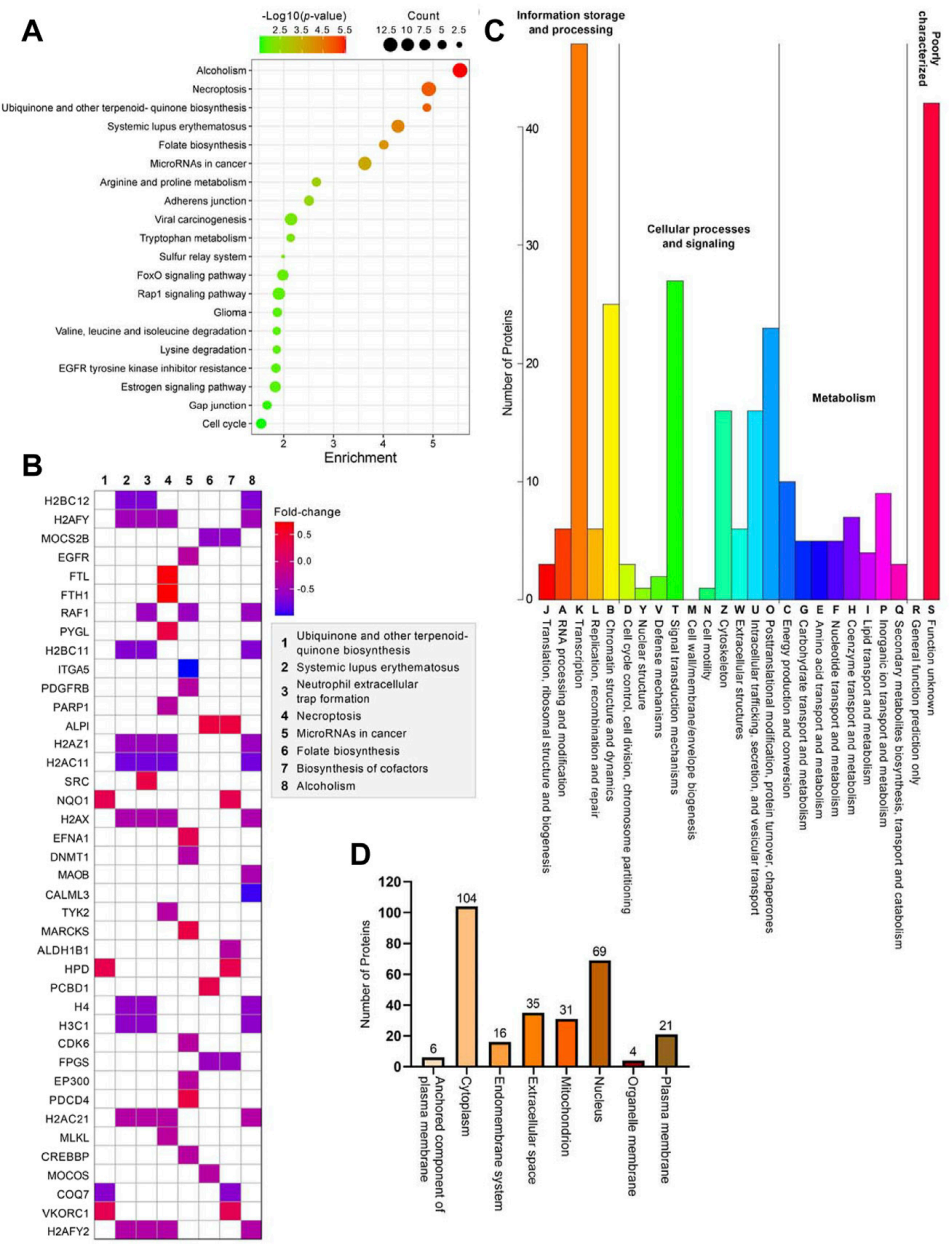
Bioinformatics analyses of 286 differentially expressed proteins identified in the ZIP8-KO cell model. **(A)** Bubble plot of KEGG pathway enrichment analysis using KEGGPATHWAY database. **(B)** Heatmap of genes and their relative pathways analyzed using KEGG pathway enrichment analysis. **(C)** KOG annotation analysis using eggNOG database. **(D)** Main subcellular distribution of the differentially expressed proteins analyzed using BUSCA.

Further bioinformatics analyses were carried out on the differentially expressed proteins to better resolve the affected molecular pathways or biological systems in cells without the ZIP8 protein. Briefly, KEGG enrichment analysis indicated that alcoholism, necroptosis, biosynthesis of cofactors (e.g., ubiquinone), systemic lupus erythematosus, folate biosynthesis, microRNAs in cancer were among the most significantly enriched pathways (Figures 3A,B; Supplementary Table S5). In addition, other KEGG pathways that might also involve in ZIP8-KO including those that were related to viral carcinogenesis, EGFR tyrosine kinase inhibitor resistance, degradation of lysine, valine, leucine, and isoleucine, and signaling of FoxO, Rap1, and estrogen (Figure 3A).

KOG annotation analysis revealed that the pathways of transcription, chromatin structure and dynamics, signal transduction mechanisms, posttranslational modification, protein turnover, and chaperones encompassing the highest numbers of differentially expressed proteins, whereas those involved in nuclear structure, cell motility, and cell wall/membrane/envelope biogenesis contained little to no proteins (Figure 3C; Supplementary Table S6). Most of the differentially expressed proteins were proteins mainly located in the cytoplasm, followed by the nucleus (Figure 3D; Supplementary Table S7). Only 6 (ALPI, CD109, CPM, EFNA1, NEGR1, and TCTN3), 4 (CCNB1, CISD1, MPC1, and MRS2), and 21 (ABCG2, ANPEP, ATP11A, ATP13A1, CD83, CD9, CEMIP2, CSPG4, DNAJB11, EGFR, HLA-B, ITGA5, ITGB1, PCSK9, PDGFRB, PTPRF, SCAMP1, SLC7A2, TFRC, TGFBR1, and TSC2) proteins were located in the anchored component of the plasma membrane, organelle membrane, and plasma membrane, respectively (Figure 3D).

### PPI network analysis of differentially expressed proteins

**FIGURE 4:**
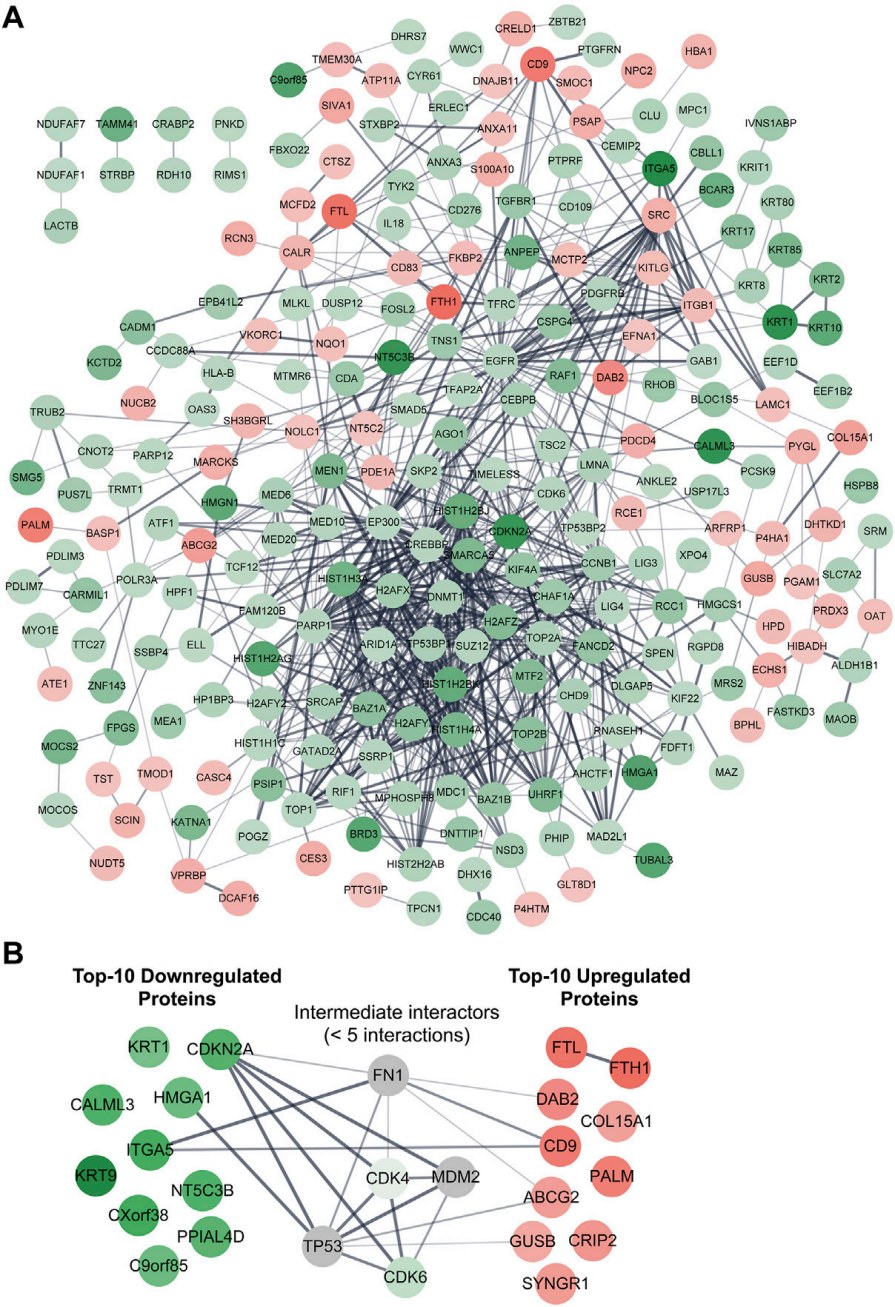
PPI network analysis of differentially expressed proteins identified in the ZIP8-KO cell model. Functional and physical interactions of all 286 **(A)** or 20 top-10 upregulated/downregulated **(B)** proteins were subjected to STRING analysis. Disconnected proteins are excluded in the network of **(A)** but included in **(B)**. Proteins within five interactions (max number of interactors to show = <5) are included in **(B)**. The thicker the line between two connected proteins indicates a higher confidence level of the interaction. Downregulated and upregulated proteins are highlighted in green and red, respectively; proteins highlighted in grey represent proteins that are not differentially expressed.

We then assessed the PPIs among all the 286 differentially expressed proteins, and the results indicated that a total of 819 interactions were generated from 243 proteins (Figure 4A). The remaining 43 proteins, including three uncharacterized proteins (C1orf198, C17orf75, and CXorf38), did not directly interact with any of the analyzed proteins (not displayed in Figure 4A). However, another uncharacterized protein, C9orf85, was shown to interact with TMEM30A (Figure 4A). Furthermore, we found that a few proteins were able to interact with more than 30 different proteins, and these “super interactors” were CREBBP, EP300, EGFR, H2AFZ, H2AFX, PARP1, SMARCA5, and SRC (Figure 4A). On the other hand, we checked if ZIP8 could directly interact with any of the differentially expressed proteins and found that ZIP8 only interacted with one protein, the transferrin receptor protein 1 (TFRC) (data not shown). In addition, proteins that could interact directly with the four uncharacterized proteins, C1orf198, C9orf85, C17orf75, and CXorf38, were examined and shown in Supplementary Figure S3.

### Top-10 upregulated and downregulated proteins associated with ZIP8-KO human cells

We then retrieved the proteins with the most significant changes in their expression based on fold-change values (*p* ≤ 0.05). The top-10 most upregulated proteins, from highest to lowest, were FTH1, FTL, CD9, PALM, DAB2, CRIP2, SYNGR1, ABCG2, COL15A1, and GUSB (Table 1). On the other hand, the top-10 most downregulated proteins, from lowest to highest, were KRT9, CXorf38, ITGA5, KRT1, CALML3, NT5C3B, CDKN2A, PPIAL4D, C9orf85, and HMGA1 (Table 2). Notably, two of the four uncharacterized proteins (C9orf85 and CXorf38) were among the top-10 most downregulated proteins.

**TABLE 1:** Top-10 upregulated proteins identified in the ZIP8-KO cells.

Protein (UniProt ID)	Protein name	Fold-change	*p*-value	Description
FTH1 (P02794)	Ferritin heavy chain	1.6552	0.0000	• Stores iron in a soluble, non-toxic, readily available form • Important for iron homeostasis shows ferroxidase activity
FTL (P02792)	Ferritin light chain	1.6216	0.0000	• Stores iron in a soluble, non-toxic, readily available form • Important for iron homeostasis
CD9 (P21926)	CD9 antigen	1.5677	0.0014	• Involves in cell adhesion, cell motility, and tumor metastasis
PALM (O75781)	Paralemmin-1	1.5455	0.0007	• Involves in plasma membrane dynamics and cell process formation
DAB2 (P98082)	Disabled homolog 2	1.4864	0.0000	• Expression is downregulated in numerous aggressive cancers (e.g., prostate cancer and medulloblastoma) • Involves in several processes such as innate immune response, inflammation and cell growth inhibition, apoptosis, cell survival, angiogenesis, cell migration, and maturation • Induces G0/G1 cell cycle arrest and promotes apoptosis
CRIP2 (P52943)	Cysteine-rich protein 2	1.4234	0.0001	• Zinc ion binding
SYNGR1 (O43759)	Synaptogyrin-1	1.3960	0.0001	• Regulates exocytosis
ABCG2 (Q9UNQ0)	Broad substrate specificity ATP-binding cassette transporter ABCG2	1.3884	0.0026	• Regulates multidrug resistance
				• Acts as human drug efflux transporter
COL15A1 (P39059)	Collagen alpha-1 (XV) chain	1.3633	0.0001	• Structural protein that stabilizes microvessels and muscle cells, both in the heart and in skeletal muscle • Associates with drug resistance
GUSB (P08236)	Beta-glucuronidase	1.3351	0.0010	• Degradation of dermatan and keratan sulfates

**TABLE 2:** Top-10 downregulated proteins identified in the ZIP8-KO cells.

Protein (UniProt ID)	Protein name	Fold-change	*p*-value	Description
KRT9 (P35527)	Keratin, type I cytoskeletal 9	−2.4219	0.0001	• Involves in keratin filament assembly • A cytoskeleton intermediate filament protein
CXorf38 (Q8TB03)	Uncharacterized protein CXorf38	−1.9912	0.0397	• Unknown biological function
ITGA5 (P08648)	Integrin alpha-5	−1.9599	0.0004	• Belongs to the integrin alpha chain family • Vital for promoting cancer cell invasion and metastasis
KRT1 (P04264)	Keratin, type II cytoskeletal 1	−1.9039	0.0003	• May regulate the activity of kinases such as PKC and SRC *via* binding to integrin beta-1 (ITB1) and the receptor of activated protein C kinase 1 (RACK1)
CALML3 (P27482)	Calmodulin-like protein 3	−1.8770	0.0375	• May function as a specific light chain of unconventional myosin-10 (MYO10) • Enhances MYO10 translation, possibly by acting as a chaperone for the emerging MYO10 heavy chain protein • May compete with calmodulin by binding, with different affinities, to cellular substrates • Promotes JNK1/2 and ERK1/2 pathway
NT5C3B (Q969T7)	7-methylguanosine phosphate-specific 5′-nucleotidase	−1.8717	0.0336	• Plays a role in inflammation and tissue remodeling • May result in airway wall thickening
CDKN2A (Q8N726)	Tumor suppressor ARF	−1.8563	0.0044	• Acts as a negative regulator of the proliferation of normal cells by interacting strongly with CDK4 and CDK6, inhibiting their ability to interact with cyclins D • A tumor suppressor gene
PPIAL4D (F5H284)	Peptidyl-prolyl cis-trans isomerase A-like 4D	−1.8258	0.0018	• Accelerates the folding of proteins • Catalyzes the cis-trans isomerization of proline imidic peptide bonds in oligopeptides
C9orf85 (Q96MD7)	Uncharacterized protein C9orf85	−1.7043	0.0005	• Unknown biological function
HMGA1 (P17096)	High mobility group protein HMG-I/HMG-Y	−1.6915	0.0010	• A coactivator and coregulator of transcriptional activity

We also performed a PPI network analysis on the top-10 upregulated and downregulated proteins, and the results indicated that these proteins generally did not interact with each other, with the exception of FTL–FTH1 and ITGA5–CD9 (Figure 4B). When proteins within five interactions were included in the PPI network analysis, five proteins (CDK4, CDK6, FN1, MDM2, and TP53) were identified as the “intermediate interactors” for some of the analyzed proteins (Figure 4B).

### Relationships between essential micronutrients levels and uncharacterized proteins C9orf85 and CXorf38

**FIGURE 5:**
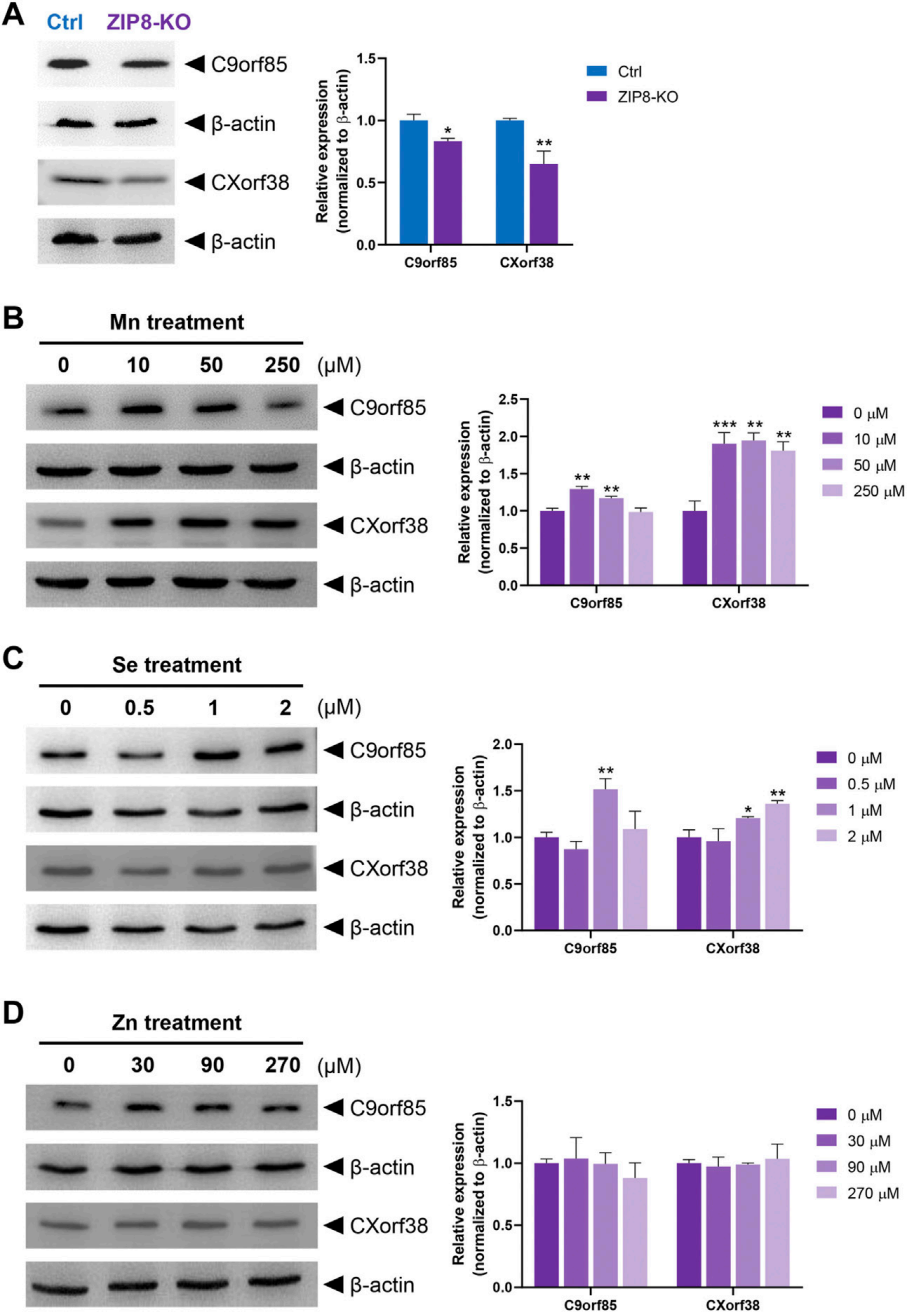
Immunoblot analysis of uncharacterized human proteins C9orf85 and CXorf38 treated with essential micronutrients (see Supplementary Figure S4 for full blot images). **(A)** Protein levels of C9orf85 and CXorf38 in HeLa parental cells (with WT ZIP8) and ZIP8-KO HeLa cells. **(B–D)** Protein levels of C9orf85 and CXorf38 in ZIP8-KO HeLa cells exposed to various concentrations of essential micronutrients Mn **(B)**, Se **(C)**, and Zn **(D)** for 24 h. Images are representative of independent experiments with similar expression trends of at least three independent experiments, and error bars represent mean ± SD of triplicate samples. *p ≤ 0.05; **p ≤ 0.01; ***p ≤ 0.001.

Next, we investigated the potential role of micronutrients on the expressions of C9orf85 and CXorf38. Immunoblot analysis was performed to verify the iTRAQ data, and the results indicated that the protein levels of C9orf85 and CXorf38 were indeed lower in the ZIP8-KO cells compared with the HeLa parental cells (Figure 5A; Supplementary Figure S4). Then, ZIP8-KO cells were treated with various concentrations of micronutrients (Mn, Se, and Zn), and the expressions of C9orf85 and CXorf38 were assessed. Overall, we found that the expression of C9orf85 could be induced only briefly by Mn and Se, and it could not be influenced by Zn (Figures 5B–D). However, for CXorf38, its expression could be induced effectively by Mn (Figure 5B), not so effectively by Se (Figure 5C), and not induced by Zn (Figure 5D). These findings suggest that the downregulation of C9orf85 and CXorf38 may be associated with the dysregulation of intracellular micronutrient levels in the ZIP8-KO cells.

### Co-expression of ZIP8 and Top-10 upregulated or downregulated ZIP8-KO-associated protein genes in cancers

**FIGURE 6:**
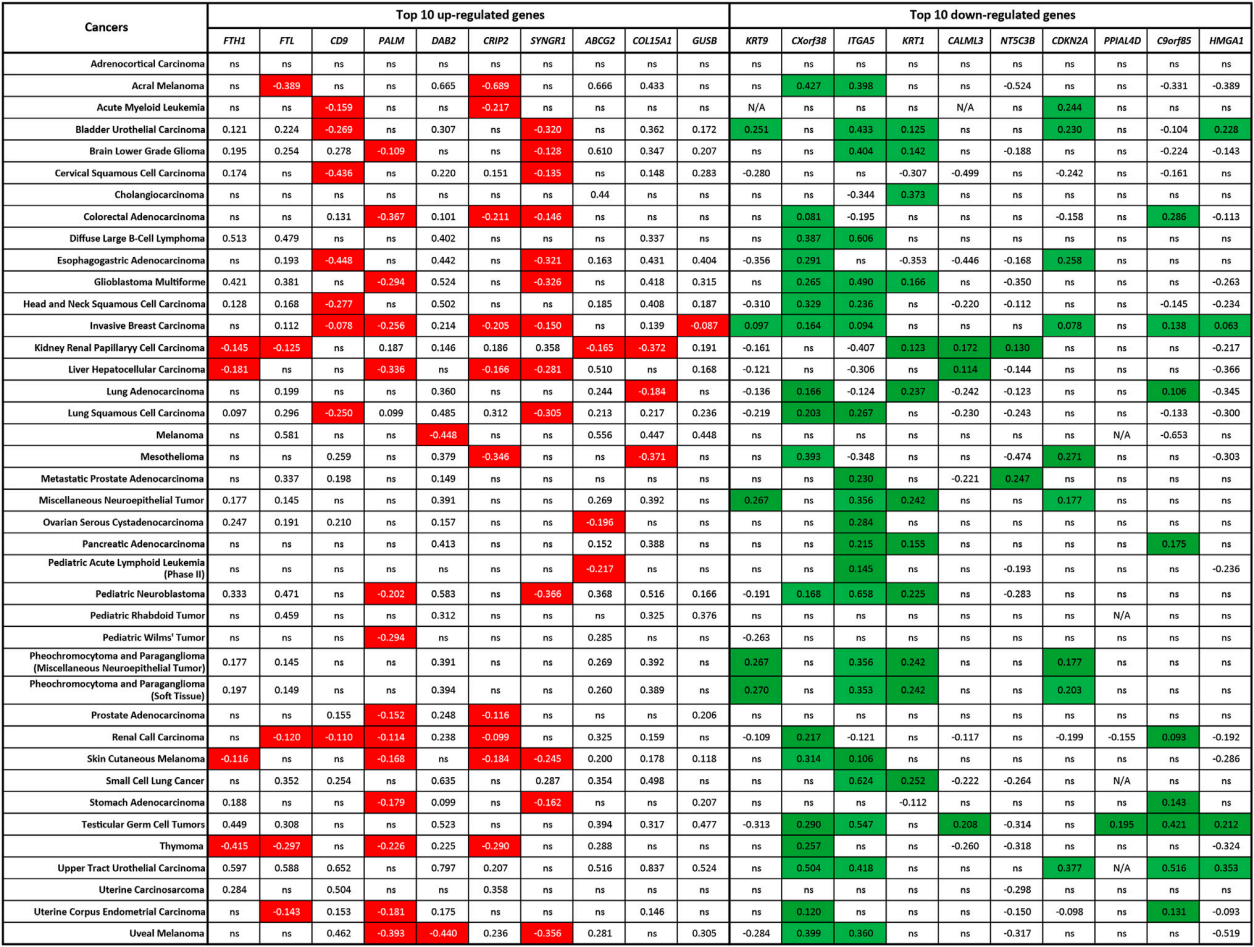
Co-expression of *ZIP8* (*SLC39A8*) and top-10 upregulated or downregulated ZIP8-KO-associated protein genes in 40 cancer types. Clinical data were obtained from TCGA database and analyzed by Spearman’s correlation. A Spearman’s correlation value greater than 0 indicates a positive correlation, and a value less than 0 indicates a negative correlation. Co-expressions that signify the proteome of ZIP8-KO were highlighted: negative correlations between the *ZIP8* and upregulated protein genes (highlighted in red) or positive correlations between the *ZIP8* and downregulated protein genes (highlighted in green). Only values with *p* ≤ 0.05 were shown. N/A: data not available; ns: non-significant.

Lastly, we evaluated the possible connections between the gene expressions of *ZIP8* and the top-10 upregulated or downregulated ZIP8-KO-associated protein genes in a range of cancers using the TCGA database. We have previously shown that upregulated *ZIP8* (*SLC39A8*) gene expression was common across multiple cancer types (Liang et al., 2021). Here, to determine if the expression of *ZIP8* is significantly correlated to the gene expressions of the top-10 upregulated or downregulated proteins in the ZIP8-KO cells, the following criteria must be met: a negative correlation between the *ZIP8* and an upregulated protein gene; or a positive correlation between the *ZIP8* and a downregulated protein gene. Based on the above criteria, we identified ZIP8-KO-associated protein genes that were significantly co-expressed with *ZIP8* in different cancer types (Figure 6). Specifically, *CXorf38* was significantly co-expressed with *ZIP8* in 18 out of 40 cancer types, whereas *C9orf85* was only in nine out of 40 cancer types (Figure 6).

## Discussion

Recent advances in omics technologies have enabled us to rapidly investigate the status of the biomolecules of interest in a selected biological system (Tan et al., 2018). Single-gene KO cell lines are useful models to study the function of a particular gene, and when an omics analysis (SGKOmics analysis) is performed on such cell models, the overall biophysiological effects of the gene on the entire cell can be revealed.

The ZIP8 is a transmembrane protein that involves in the uptake of Fe, Mn, Zn, and Se, and therefore, cells with impaired ZIP8 are often associated with dysregulated intracellular levels of these essential micronutrients (McDermott et al., 2016; Zang et al., 2016; Lin et al., 2017; Liang et al., 2021). Although studies have indicated that the expression of ZIP8 can be affected by different physiological conditions (e.g., during lactation, Cd exposure, ZnO nanoparticle treatment, and inflammation) (Kelleher et al., 2012; Gao et al., 2017; Xu et al., 2017; Pan et al., 2020; Hatano et al., 2021), it is largely unclear how impaired ZIP8 will affect the cells other than having an impact on the intracellular homeostasis of micronutrients.

Here, using an iTRAQ-based quantitative proteomics approach, the proteome of a ZIP8-KO cell model was examined for the first time. This ZIP8-KO stable cell line was previously established from the HeLa cells using the CRISPR/Cas9 genome editing technology (Liang et al., 2021). An endogenous KO of the *ZIP8* gene could allow us to study the functions of this gene more explicitly by mimicking the conditions of patients with non-functional ZIP8—the physiological roles of ZIP8 in relation to a range of human diseases have been extensively reviewed (Zang et al., 2016; Fujishiro and Himeno, 2019; Nebert and Liu, 2019). However, only a ZIP8-KO cell model cannot fully demonstrate or explain the connections between ZIP8 and human diseases. Thus, further research should be carried out on additional ZIP8-KO cell and animal models, as well as patient samples with impaired ZIP8.

Bioinformatics analysis of the proteomic changes in the ZIP8-KO cells revealed proteins and their associated pathways that were potentially affected by the dysregulation of intracellular micronutrient levels, including a range of signaling pathways that are long-known for their roles in human diseases. For example, the FoxO-related signaling pathway is involved in sarcopenia (a skeletal muscle disorder) and cancer development, especially upon nutrient starvation (Jiramongkol and Lam, 2020; Liu et al., 2021); Signaling pathway of estrogen is highly regulated by the nutrient availability, and the abnormal actions of estrogen can lead to various metabolic syndromes as well as affect the immune and inflammatory conditions of the cells (Mauvais-Jarvis et al., 2013); Notch-related signaling pathway is involved in the regulation of neurogenesis and central nervous system, which is linked to neurological disorders (e.g., Parkinson’s disease and Alzheimer’s disease) and neuroendocrine tumors (e.g., glioma) (Kim et al., 2020; Zhou et al., 2022).

In the ZIP8-KO cells, it was shown that many of the differentially expressed proteins were viral infection-related, as pathways such as viral RNA replication, integration, carcinogenesis, and receptor activity were significantly enriched. Evidence is clear that an imbalanced level of intracellular micronutrients can disrupt normal immune function and increase the risk of microbial infection (Gombart et al., 2020). It has also been suggested that ZIP8 is one of the key proteins in the pathogenesis of viral infections, especially during respiratory viral infections, since ZIP8 is highly expressed in the human lungs (Sadeghsoltani et al., 2022). Among the ZIP8-transportable essential micronutrients, Zn and Se are the two main elements that exhibit antiviral properties and play vital roles in the immune response against viral infections (Kar et al., 2019; Tan et al., 2020). In addition to preventing viral infection, Se and many Se compounds are also potent anticancer agents that can be utilized in cancer prevention and therapy (Tan et al., 2019). Furthermore, previous studies have suggested that ZIP8 is associated with the development of cancers, probably due to disrupted Se homeostasis (Liu et al., 2018; Liang et al., 2021). However, the intertwining relationships between the differentially expressed proteins identified in the ZIP8-KO cells, intracellular micronutrient levels, and human diseases have remained unclear and warrant further studies.

Humans have approximately 22,000 protein-coding genes, which could be translated into hundreds of thousands of proteins through alternative splicing (Wilhelm et al., 2014). To date, the characteristics of many proteins have remained uninvestigated, and they are collectively known as the “uncharacterized” protein and commonly named as the “open reading frame (ORF)” proteins (Duek and Lane, 2019). Early studies of uncharacterized proteins usually focused only on whether the atypical ORF regions found within the genome could be translated into proteins. Further research on uncharacterized proteins, especially those encoded by short-ORFs, showed that they were indeed involved in diverse biological processes, including but not limited to cell proliferation (Galindo et al., 2007), DNA damage repair (Slavoff et al., 2014), muscle activities (Anderson et al., 2015; Nelson et al., 2016), cell signaling (Chng et al., 2013), mRNA degradation (D'Lima et al., 2017), and phagocytosis (Pueyo et al., 2016). Therefore, uncharacterized proteins have attracted increasing attention in recent years, but systemic research on these proteins is still lacking (Delcourt et al., 2018; Wu et al., 2022).

In the current study, one of the most interesting findings in the altered proteome of ZIP8-KO cells was the discovery of two uncharacterized proteins among the top-10 significantly downregulated proteins: C9orf85 and CXorf38. Although we did not test the potential biological functions of these two uncharacterized proteins, we did find that their expressions could be induced by either Mn or Se, thus unveiling the possible roles of these proteins in nutrition. These findings also indicate that C9orf85 and CXorf38 have the potential to be targeted as biomarkers and/or therapeutic targets for micronutrient deficiency diseases. Nevertheless, further studies are required to investigate the functions of these two uncharacterized proteins in human diseases.

## Conclusion

To conclude, this study shows that a functional ZIP8 is important in maintaining normal cellular physiology. In particular, the differentially expressed proteins (e.g., C9orf85 and CXorf38) identified in the ZIP8-KO cells could be potential targets for diagnosing and/or treating a wide range of human ZIP8-associated diseases, including but not limited to malnutrition, viral infection, and cancers.

## Data Availability

The datasets presented in this study can be found in online repositories. The names of the repository/repositories and accession number(s) can be found below: http://www.proteomexchange.org/, PXD036680.

## Supplementary material

SUPPLEMENTARY TABLE S1 Data of iTRAQ-coupled LC-MS/MS analysis.

SUPPLEMENTARY TABLE S2 Data of gene ontology (biological process) enrichment analysis of differentially expressed proteins.

SUPPLEMENTARY TABLE S3 Data of gene ontology (cellular component) enrichment analysis of differentially expressed proteins.

SUPPLEMENTARY TABLE S4 Data of gene ontology (molecular function) enrichment analysis of differentially expressed proteins.

SUPPLEMENTARY TABLE S5 Data of Kyoto encyclopedia of genes and genomes pathway enrichment analysis of differentially expressed proteins.

SUPPLEMENTARY TABLE S6 Data of eukaryotic orthologous groups annotation analysis of differentially expressed proteins.

SUPPLEMENTARY TABLE S7 Data of subcellular location of differentially expressed proteins.

SUPPLEMENTARY FIGURE S1 Heatmap of identified proteins in the ZIP8-WT or ZIP8-KO HeLa cells based on hierarchical cluster analysis of individual samples **(A)** or combined samples **(B)**.

SUPPLEMENTARY FIGURE S2 Significantly enriched Gene Ontology (GO) categories **(A)** and differentially expressed proteins **(B)** related to “transporter regulation”, “metal response”, and “stress response.” BP: Biological process; MF: Molecular function.

SUPPLEMENTARY FIGURE S3 PPI network analysis of uncharacterized proteins C1orf198, C9orf85, C17orf75, and CXorf38.

SUPPLEMENTARY FIGURE S4 Immunoblotting of HeLa parental cells (with ZIP8-WT) or ZIP8-KO cells probed with C9orf85 (PA5-65639; Invitrogen), CXorf38 (PA5-62139; Invitrogen), or β-actin (A5441; Sigma-Aldrich) antibody, displayed in full blots.
