# Supplementary figures and images for: Single-gene knockout-coupled omics analysis identifies C9orf85 and CXorf38 as two uncharacterized human proteins associated with ZIP8 malfunction
Source: Front Mol Biosci. 2022 Oct 18;9:991308. doi: 10.3389/fmolb.2022.991308 (PMC9623088; doi:10.3389/fmolb.2022.991308)

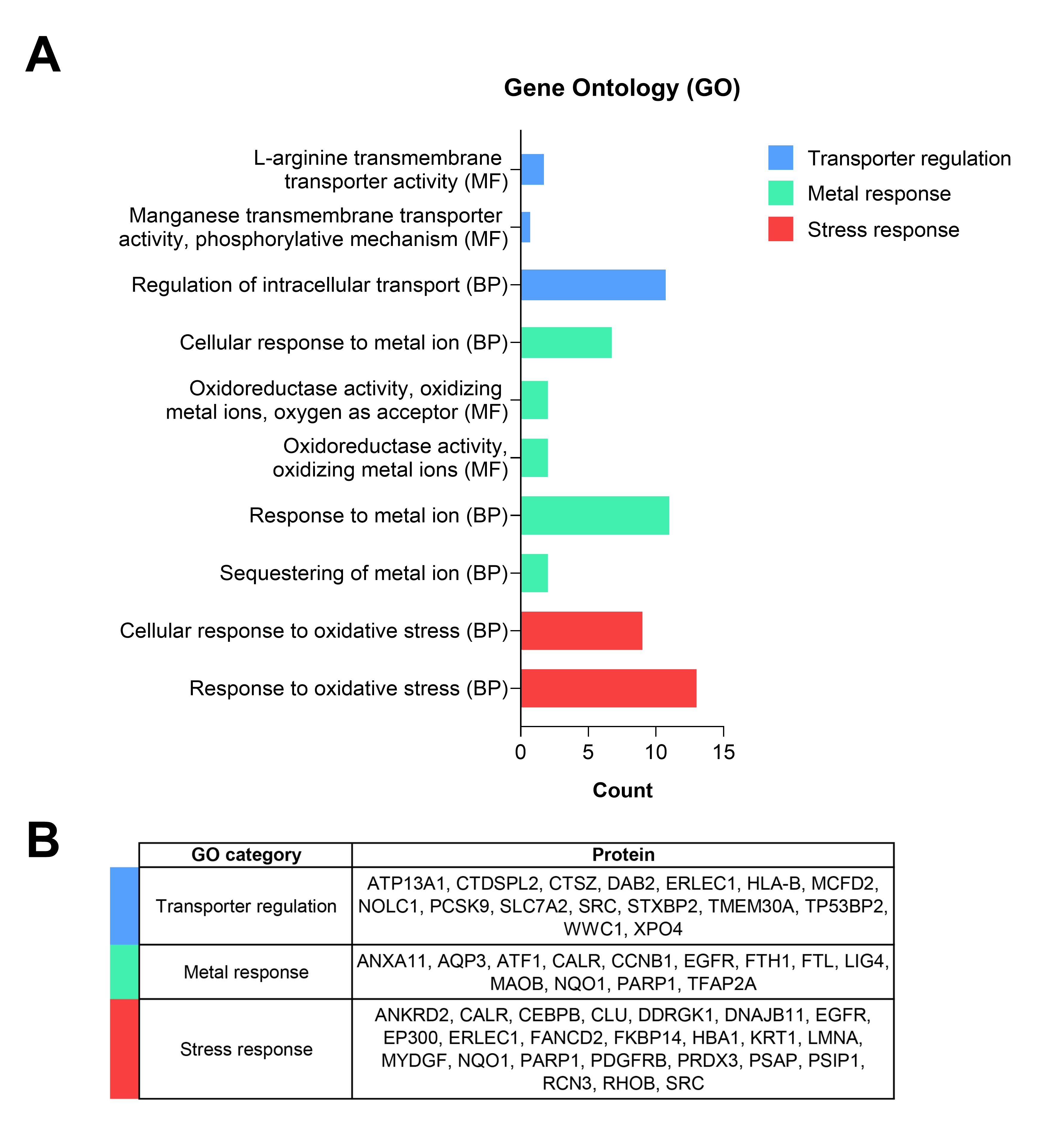

Supplement: Supplementary file 5 [file Image2.TIF]
